# Revealing the biological mechanism of acupuncture in alleviating excessive inflammatory responses and organ damage in sepsis: a systematic review

**DOI:** 10.3389/fimmu.2023.1242640

**Published:** 2023-09-11

**Authors:** Lin Yang, Dan Zhou, Jiaojiao Cao, Fangyuan Shi, Jiaming Zeng, Siqi Zhang, Guorui Yan, Zhihan Chen, Bo Chen, Yi Guo, Xiaowei Lin

**Affiliations:** ^1^ School of Acupuncture-Moxibustion and Tuina, Tianjin University of Traditional Chinese Medicine, Tianjin, China; ^2^ Research Center of Experimental Acupuncture Science, Tianjin University of Traditional Chinese Medicine, Tianjin, China; ^3^ Ministry of Education, State Key Laboratory of Component-Based Chinese Medicine, Tianjin University of Traditional Chinese Medicine, Tianjin, China; ^4^ The First Teaching Hospital of Tianjin University of Traditional Chinese Medicine, Pharmacy Department, Tianjin, China; ^5^ Tianjin Key Laboratory of Modern Chinese Medicine Theory of Innovation and Application, Tianjin University of Traditional Chinese Medicine, Tianjin, China; ^6^ School of Traditional Chinese Medicine, Tianjin University of Traditional Chinese Medicine, Tianjin, China

**Keywords:** sepsis, acupuncture, anti-inflammation, autonomic nerve, mechanism research

## Abstract

Sepsis is a systemic inflammation caused by a maladjusted host response to infection. In severe cases, it can cause multiple organ dysfunction syndrome (MODS) and even endanger life. Acupuncture is widely accepted and applied in the treatment of sepsis, and breakthroughs have been made regarding its mechanism of action in recent years. In this review, we systematically discuss the current clinical applications of acupuncture in the treatment of sepsis and focus on the mechanisms of acupuncture in animal models of systemic inflammation. In clinical research, acupuncture can not only effectively inhibit excessive inflammatory reactions but also improve the immunosuppressive state of patients with sepsis, thus maintaining immune homeostasis. Mechanistically, a change in the acupoint microenvironment is the initial response link for acupuncture to take effect, whereas PROKR2 neurons, high-threshold thin nerve fibres, cannabinoid CB2 receptor (CB2R) activation, and Ca^2+^ influx are the key material bases. The cholinergic anti-inflammatory pathway of the vagus nervous system, the adrenal dopamine anti-inflammatory pathway, and the sympathetic nervous system are key to the transmission of acupuncture information and the inhibition of systemic inflammation. In MODS, acupuncture protects against septic organ damage by inhibiting excessive inflammatory reactions, resisting oxidative stress, protecting mitochondrial function, and reducing apoptosis and tissue or organ damage.

## 1 Introduction

The definition of sepsis has evolved, deepened, and improved with the development of medical practices. In Sepsis 3.0, sepsis is defined as life-threatening organ dysfunction due to an imbalance in the host inflammatory response caused by infection (1); reducing inflammation and correcting organ dysfunction are the core strategies for treating sepsis. Sepsis is a critical disease with high incidence and rapid development. It is a prominent problem and a key disease in contemporary medicine. The latest data from the *Lancet* in 2020 show that in 2017, there were approximately 48.9 million cases of sepsis worldwide and approximately 11 million sepsis-related deaths, accounting for 19.7% of the total deaths worldwide (2). More importantly, these figures may be further exacerbated by the global COVID-19 pandemic and high medical costs associated with sepsis. Modern research has shown that early intervention in patients with possible sepsis, extensive cooperation in the medical field, and optimisation and improvement of treatment plans are effective ways to reduce the occurrence of sepsis.

Currently, the treatment strategies for sepsis include anti-infection and organ support strategies. Acupuncture has been used to treat inflammatory diseases for thousands of years (3, 4). Modern evidence has shown the significant anti-inflammatory effects of acupuncture on sepsis. Acupuncture may be a promising complementary strategy for early prevention and treatment of septic inflammation, improvement of survival rates, and protection of organs. Therefore, we summarised the pathological process of sepsis, current status of the clinical application of acupuncture in treating sepsis, commonly used animal models for the study of the mechanism of acupuncture in treating sepsis, and acupuncture intervention parameters, focusing on the research progress of the mechanism of acupuncture prevention and treatment of sepsis from the perspectives of the acupoint microenvironment, autonomic neurobiological mechanisms, and target organ effects.

## 2 Pathological mechanism of sepsis

### 2.1 Hyperinflammatory response

**Figure 1:**
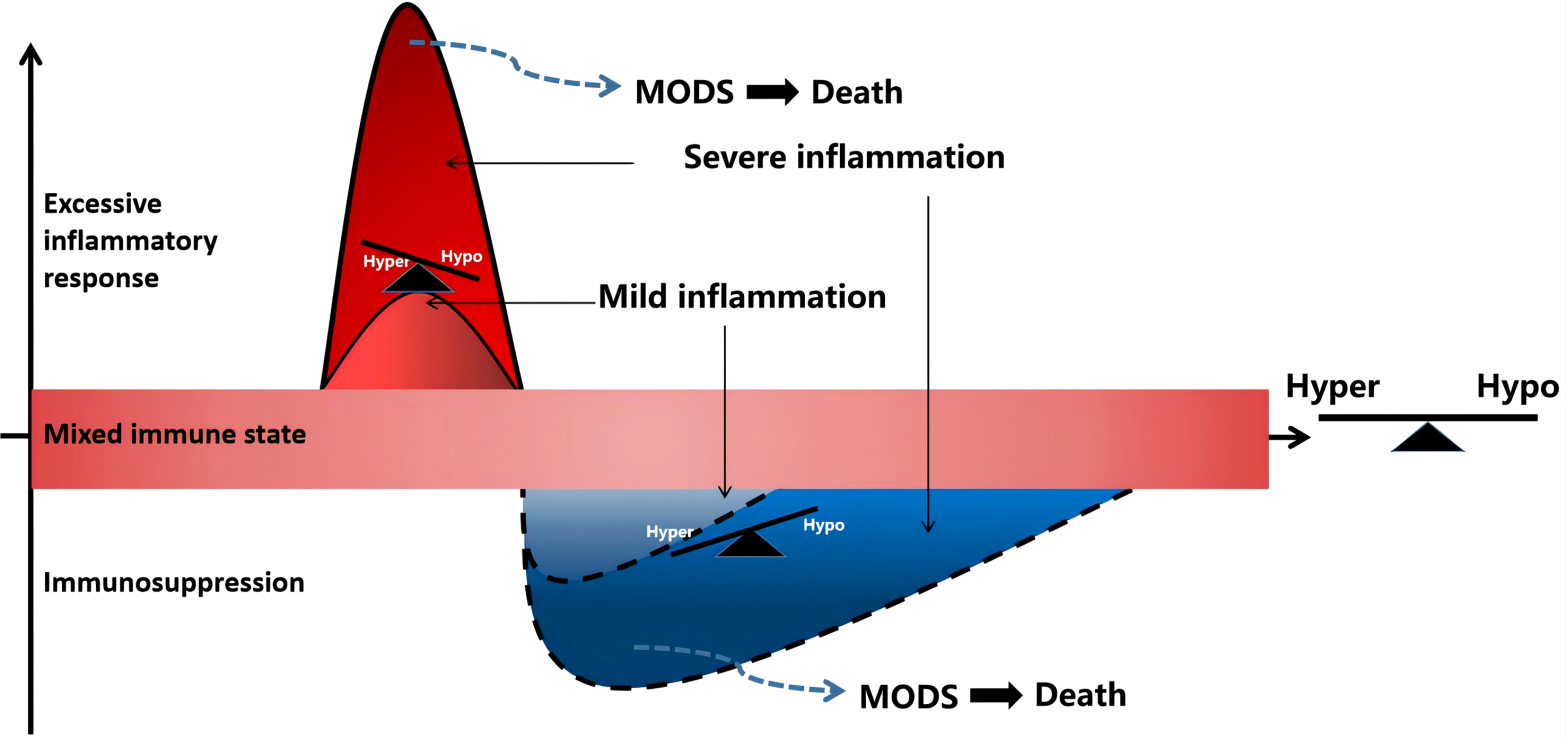
Sepsis is a process of inflammatory imbalance. multiple organ dysfunction syndrome (MODS), multiple organ dysfunction syndrome; Hyper, hyper-inflammatory; hypo-inflammatory.

Sepsis is a gradual sequential reaction. Starting with an inflammatory reaction, the body can exhibit three states when stimulated by pathogens. First, when inflammatory cells are overactivated, causing an imbalance between proinflammatory and anti-inflammatory effects, an uncontrolled inflammatory cascade reaction is initiated. Uncontrolled inflammatory reactions in the body are considered an important basis for multiple organ dysfunction caused by sepsis, which is mainly characterized by the release of a large number of proinflammatory mediators. Second, if sepsis cannot be controlled for a short time, a mixed immune state (i.e., an immune disorder) can occur. Finally, as the disease progresses, the immune state gradually transforms into immunosuppression/immune paralysis, causing repeated infections (**Figure 1**). In the early stage of sepsis, the first death peak is caused by multiple organ failure caused by uncontrolled inflammation storms, whereas the second death peak of sepsis patients is often caused by secondary severe infection as a result of immunosuppression.

The cytokine storm stage is a period with a high incidence of death in patients with sepsis. During uncontrolled inflammation, excessive activation of inflammatory cells and the release of a large number of inflammatory factors are important mechanisms of multiple organ dysfunction in sepsis. Pathogen-associated molecular patterns (PAMPs) and damage-associated molecular patterns (DAMPs) are recognized and activated by pattern recognition receptors (PRRs) on immune cell surfaces. When the number of pathogens is limited, local inflammatory reactions are sufficient to remove them. For example, macrophages engulf bacteria and produce a series of inflammatory cytokines that activate the innate immune system to fight pathogenic invasion. However, when a large number of pathogens invade, immune cells are overactivated, resulting in the release of numerous inflammatory factors, such as tumor necrosis factor-α (TNF-α), interleukin-1beta (IL-1beta), IL-6, and IL-17, which form a storm of inflammatory factors and initiate a sepsis-like network reaction. During sepsis development, the body is not constantly in a state of immune activation. Anti-inflammatory and proinflammatory responses occur almost simultaneously, and anti-inflammatory cytokines limit persistent or excessive inflammatory responses. The negative feedback regulation mechanism of the body’s immune system can promote the secretion of anti-inflammatory cytokines, such as IL-4, IL-10, and transforming growth factor beta (TGF-beta), thereby inhibiting the production and release of proinflammatory mediators, such as TNF-α, IL-1β, and IL-6. However, the amount of proinflammatory cytokines and anti-inflammatory cytokines in patients with sepsis is often unbalanced. When anti-inflammatory cytokines are insufficient, the body experiences immune dysfunction, which aggravates the progression of sepsis. TNF-α is the earliest inflammatory factor synthesized during the occurrence and development of sepsis, and also one of the initiating factors of sepsis (5). Recent research has shown that the gene polypeptide at the promoter-308 site is closely related to the susceptibility and outcome of sepsis and septic shock (6). It was found that the G/A gene polymorphism of TNF-α-308 is significantly positively associated with a higher incidence of sepsis and increased expression of cytokines such as TNF-α, IL-6, and IL-8 in patients (7). In addition, the study found that subjects with IL-10-1082 AA and IL-6-174 CC genotypes had a higher risk of sepsis and increased mRNA levels. Overproduction of IL-10 can lead to a compensatory anti-inflammatory response and inhibit the inflammatory defense system in patients with sepsis. IL-6 is the main mediator of systemic inflammatory response syndromes. Excess IL-6 activates the coagulation system and increases vascular permeability, providing conditions for rapid diffusion of inflammation. Therefore, these genetic variants could be used as therapeutic targets in patients with sepsis (8).

### 2.2 Immunosuppression

If sepsis is not controlled within a short time, a mixed immune state, namely an immune disorder, can occur. During the period of mixed immunity, the cytokine cascade amplification reaction in the high inflammatory response state of the body begins to decrease, and the compensatory anti-inflammatory reaction initiated by the inflammatory reaction continues. In the late stage of mixed immunity, there are usually three outcome trends (1): the immune state of the body gradually returns to balance and the disease improves; (2) mixed immune state: the inflammatory and anti-inflammatory mechanisms of the body are not reconciled, and the clinical manifestations are complex; (3) immune suppression: there is severe immune damage caused by strong pathogenic factors or deterioration due to the disease, and the immune state gradually transforms into immunosuppression or loss of immune ability, also known as immune paralysis.

Immunosuppression in the late stages of sepsis is the main cause of long-term complications and death from sepsis. Currently, immunosuppression is believed to be mainly related to anti-inflammatory mediators and apoptosis. Immunosuppression involves abundant lymphocyte apoptosis accompanied by a decrease in their proliferative capacity, leading to a decrease or no reaction of T cells, presenting an immune response dominated by type 2 helper T (Th2) cells, a decrease in the number of CD4+ antigen-presenting cells, apoptosis or incompetence, and an inability to present antigens and produce effectors (9). Specifically, the anti-inflammatory factors produced by monocytes significantly increased, such as IL-10 and TGF-β, and proinflammatory factors (such as TNF-α and IL-1β) decreased, presenting immunosuppression. In addition, the production of Th2 type cytokines (IL-4, IL-10) is increased and Th1 type cytokines (IL-12 and IFN-γ) is decreased, which affects the differentiation of T lymphocytes and causes the body to present a Th2-based immune response and damage the cellular immune function (10). In the immunosuppressive stage, a large number of B lymphocytes, CD4+T lymphocytes, dendritic cells, and other antigen-presenting cells undergo apoptosis, resulting in reduced antibody production and human leukocyte antigen-DR (HLA-DR) capacity (11–14). However, the number of inhibitory receptors, such as programmed cell death protein 1, regulatory T cells, and myeloid-derived suppressor cells (MDSCs), is significantly increased, leading to the development of severe immunosuppression in later stages (15). Simultaneously, neutrophils differentiate into subsets with immunosuppressive effects and produce numerous cytokines that inhibit immune response, such as IL-10 (16). In addition, inherent immune natural killer (NK) cell subsets (CD56 Hi and CD56 Low) are significantly reduced, and the degree of reduction is closely related to mortality (17).

### 2.3 Oxidative stress, mitochondrial damage, and apoptosis lead to sepsis organ damage and coagulation dysfunction

In the course of sepsis, mitochondria, which are important energy and material metabolism centers, are most vulnerable to oxidative stress damage, which is also key to organ dysfunction (18). The release of several inflammatory mediators can affect the coupling process of the normal oxidative respiratory chain in mitochondria, resulting in an increase in ROS production. The large increase in ROS damages the activity of functional enzymes and leads to the destruction of mitochondrial membrane lipids, which severely reduce the energy supply required by cells (19). This process is the main mechanism underlying oxidative stress injury caused by sepsis. Lipopolysaccharide (LPS) can increase ROS production by increasing the expression of nitric oxide synthase (NOS) and nicotinamide adenine dinucleotide phosphate (NADPH) oxidase 4 (NOX4) (20), causing oxidative stress. The accumulation of a large amount of ROS leads to insufficient ATP production in the mitochondria, and mitochondrial autophagy is a self-adaptive change in the body during inflammatory reactions. Mitochondrial autophagy can clear mitochondria with impaired function and reduce the cell damage caused by ROS during aerobic glycolysis (21). During sepsis, inflammatory reactions, oxidative stress, and mitochondrial membrane depolarization can activate a large amount of mitochondrial autophagy. Excessive mitochondrial autophagy induces apoptosis. However, when the function of phagocytes declines, apoptotic cells cannot be cleared in time, and abundant content is released, which aggravates organ damage. During sepsis, the apoptosis of intestinal epithelial cells, respiratory epithelial cells, myocardial cells, and lymphocytes increases significantly (22). Impairment of autophagy in hepatocytes in a sepsis mouse model can aggravate mitochondrial dysfunction and induce liver damage (23). Therefore, reducing ROS production, resisting oxidative stress, and reducing mitochondrial function damage and cell apoptosis are key to the treatment of multiple organ function damage in sepsis (24, 25).

Coagulation dysfunction is the main pathological manifestation of the late stages of sepsis. In mild cases, the coagulation indicators are disordered, whereas in severe cases, disseminated intravascular coagulation (DIC) may occur. The main pathological mechanisms of coagulation dysfunction caused by sepsis include activation of the coagulation system, impairment of the anticoagulation system, and inhibition of the fibrinolysis pathway. First, the stimulation of endotoxins and inflammatory mediators can damage the vascular wall, leading to the release of tissue factors (TFs) from vascular endothelial cells, neutrophils, mononuclear phagocytes, and platelets. After TFs enter the blood, they successively activate exogenous and endogenous coagulation pathways and cause extensive microvascular thrombosis through a positive feedback mechanism (26). Simultaneously, the antithrombotic microenvironment in the vascular lumen is impaired by the damage to the vascular endothelium (27). The main manifestations of anticoagulation system damage are a reduction in antithrombin level (28), damage to the protein C (PC) system (protein C, protein C inhibitor, protein S, and thrombomodulin), and damage to the tissue factor pathway inhibitor (TFPI). Clinical observations showed that a decrease in AT levels was closely related to high mortality. The PC system is the most effective physiological anticoagulant system for regulating inflammatory reactions and is mainly secreted by the liver. It inhibits the transformation of prothrombin to thrombin via negative feedback. TFPI is a natural anticoagulant and is an important exogenous inhibitor of the coagulation pathway *in vivo*. The inhibition of the fibrinolysis pathway is mainly manifested in the biphasic reaction of the fibrinolysis system, which is first activated and then inhibited. First, the tissue-type plasminogen activator (t-PA), an important physiological activator of the fibrinolysis system, can convert plasminogen into plasmin and degrade and eliminate fibrin clots. In early sepsis, TNF-α causes t-PA to be released from endothelial cells and activates the fibrinolytic system. Second, with the development of sepsis, the level of plasminogen activator inhibitor-1 (PAI-1) in the plasma continues to rise, and fibrinolytic activity is inhibited, leading to an increase in blood coagulation, and a large amount of fibrin cannot be degraded in time, leading to thrombosis (29, 30). Additionally, PAMPs and DAMPs can activate neutrophil extracellular traps (NETs), leading to platelet thrombosis and accelerated blood coagulation (31, 32). Recent research has shown that NETs can promote thrombosis by interacting with NET-carrying EVs (33).

## 3 Clinical application of acupuncture in the prevention and treatment of sepsis

Clinical evidence has accumulated regarding the extensive application of acupuncture in sepsis (**Table 1**). In clinical practice, acupuncture intervention is usually combined with conventional clinical therapies such as anti-infection, nutritional support, fluid management, and mechanical ventilation, which can significantly reduce the systemic inflammatory response and organ damage as well as improve the function of immune cells in patients with sepsis (34, 36, 38–43, 45, 46). For example, 4 Hz continuous wave electroacupuncture (EA) at the Zusanli (ST36) and Shangjuxu (ST37) acupoints combined with conventional treatment can significantly reduce plasma procalcitonin (PCT), TNF-α, intestinal fatty acid binding protein (I-FABP), and D-lactic acid levels in patients with sepsis-induced intestinal dysfunction and intestinal obstruction syndrome, and plays a protective role in intestinal function (42). The curative effect of acupuncture combined with conventional Western medicine is better than that of Western medicine alone, and can reduce inflammatory response indices and improve gastrointestinal function (40). Studies have shown that electroacupuncture or transcutaneous electrical acupoint stimulation (TEAS) combined with conventional treatment has a protective effect on intestinal function in patients with sepsis, which can effectively reduce the excretion rate of the lactulose-to-mannitol ratio (L: M) in urine and the level of serum D-lactate, and improve intestinal permeability in patients with sepsis (36, 37). Some clinical studies have shown that acupuncture combined with conventional treatment for one week can effectively reduce the levels of C-reactive protein (CRP), IL-6, and neuron-specific enolase (NSE) and improve brain damage in patients with sepsis-associated encephalopathy (SAE) (41). In addition, patients with sepsis may gradually develop rapid muscle atrophy in the intensive care unit, a phenomenon known as septic myopathy. A clinical study on the results of acupuncture treatment of sepsis-induced myopathy is underway (47). The results of this study may contribute to a new understanding of early muscle atrophy and the therapeutic effects of acupuncture in patients with sepsis-induced myopathy. These findings may provide new guidance for these patients. In conclusion, acupuncture combined with conventional treatment can significantly inhibit the excessive secretion of serum inflammatory cytokines in the early stages of sepsis, improve intestinal permeability, protect normal intestinal function, and prevent brain damage in patients with sepsis.

**Table 1:** Clinical study on acupuncture treatment of sepsis.

Sepsis model	sample size	Intervention methods	Acupoints	Parameter of acupuncture	outcome indicator	Refs.
Intestinal dysfunction	82	EA	ST36, ST37	20min, twice a day, 5 d	TNF-α↓, IL-1β↓, IAP↓	Meng. (2018) (34)
Gastrointestinal dysfunction	40	MA	EX-B2	30 min, once a day, 10 d	APACHE II scores↓, intra-abdominal pressure↓, intragastric residual volume↓	Li. (2019) (35)
AGI	49	TEAS	ST25, ST37, ST41, SP8, ST36, CV12, SP15	dilatational wave, 2 Hz/10 Hz, twice a day, 30 min	MI↑, the time of the standard of enteral nutrition and hospitalization time↓	Liu. (2020) (36)
Sepsis	50	EA	ST36, ST25, ST37, ST39	Continuous wave, 4 Hz, twice a day, 60 min, 3 d	L/M↓, serum D-lactic acid level↓	Wu. (2013) (37)
Sepsis	90	MA	ST36, GB34, PC6, RN4	the twirling reinforcing method, 30 min, once per day, 6 d	CD3+↑, CD4+↑, CD8+↑, Ig G↑, Ig A↑, Ig M↑, ICU hospitalization length↓, the hospital readmission rate and the 28-day mortality↓	Xiao. (2015) (38)
SAE	64	MA	Main acupoints: GV20, GV26, GV16, GV24, GV14, GV11	>200 r/min, 40 min, once daily, 10 d	MoCA↑, IL-6↓, CRP↓, Lac↓	Lin. (2019) (39)
Gastrointestinal dysfunction	118	MA	EX-b2, T6-T12	30 min, once a day, 10 d	WBC↓, hs-CRP↓,PCT↓, the gastrointestinal dysfunction scores↓, feeding dose↑	Li. (2019) (40)
SAE	70	EA	GV20,GV26	1 mA, 2 Hz/15 Hz, dilatational wave, 30 min every 12 hours, 7 d	CRP↓, IL-6↓, NSE↓, MoCA↑, GCS↑	Zheng. (2020) (41)
Intestinal dysfunction	71	EA	ST36,ST37	continuous wave, 4Hz, 20 min, twice a day, 5 d	PCT↓, TNF-α↓, I-FABP↓, D-lactate↓, citrulline↑	Meng. (2018) (42)
Sepsis	60	EA	ST36, RN4	continuous wave, 30 min, twice a day, 7 d	APACHE-II score↓, CD3+↑, CD4+↑, CD8+↑, CD4+/CD8+↑, HLA-DR↑	Yang. (2016) (43)
Septic shock	58	early acupoint electrical stimulation	GB30, ST32, ST36, GB39, LR3,	discontinuous wave, 2 Hz, 5 mA, 2 times daily, 7 d	MRC↑, bilateral quadriceps thickness and gastrocnemius pinnate angle↑	Wang. (2020) (44)
Sepsis	60	EA	ST36, CV4, CV6	4/20Hz, twice per day, 30 min, 5 d	sPD-1↓, CD3+T lymphocytes↑, CD4+T lymphocytes↑, NK cells↑, the percentage of lymphocytes↑, INF-γ↑, WBC↓, percentage and count of neutrophils↓, ratio of neutrophils to lymphocytes↓, CRP↓, TNF-α↓	Yang. (2022) (45)

↑, upregulated by acupuncture; ↓, downregulated by acupuncture.

EA, electroacupuncture; ST36, Zusanli; ST37, Shangjuxu; TNF-α, tumor necrosis factor-alpha; IL-1β, interleukin-1beta; IAP, intraabdominal pressure; MA, manual acupuncture; EX-B2, Jiaji; APACHE:acute physiologic and chronic health evaluation; AGI, acute gastrointestinal injury; TEAS, transcutaneous electrical acupoint stimulation; ST25, Tianshu; ST41:Jiexi; SP8:Diji; CV12, Zhongwan; SP15, Daheng; MI, antral motility index; ST39, Xiajuxu; L/M, ratio of lactulose to mannitol; GB34, Yanglingquan; PC6, Neiguan; RN4, Guanyuan; SAE, sepsis-associated encephalopathy; GV20, Baihui; GV26, Shuigou; GV16, Fengfu; GV24, Shenting; GV14, Dazhui; GV11, Shendao; MoCA, Montreal Cognitive Assessment; IL-6, interleukin 6; CRP, C-reactive protein; Lac, lactic acid; WBC, white blood cell; hs-CRP, hypersensitive C-reactive protein; PCT, procalcitonin; NSE, neuron-specific enolase; MoCA, Montreal Cognitive Assessment; GCS, Glasgow Coma Scale; I-FABP, intestinal fatty acid-binding proteins; HLA-DR, human leukocyte antigen-DR; GB30, Huantiao; ST32, Futu; GB39, Xuanzhong; LR3, Taichong; MRC, medical research council; CV4, Guanyuan; CV6, Qihai; sPD-1, soluble programmed death protein 1; INF-γ, interferon-γ.

Acupuncture not only inhibits sepsis inflammation and plays an organ-protective role but also improves the immune cell function of the body in the later stages of sepsis. Clinical studies have shown that acupuncture combined with conventional therapy can significantly reduce mortality and Acute Physiologic and Chronic Health Evaluation (APACHE) II scores in patients with sepsis. The white blood cell count, PCT, TNF-α, and IL-6 levels in blood were reduced, and CD3+, CD4+, and monocytes of HLA-DR were improved at day 7 after treatment compared with routine therapy alone (46), and the levels of T cell subsets and immunoglobulin (IgA and IgM) in the blood of sepsis patients were significantly increased (38). Human leukocyte antigen (HLA)-DR is a key effector molecule in the process of cell phagocytosis. Its reduced expression is an important indicator of immunosuppression in sepsis patients. Based on conventional treatment, EA ST36, Guanyuan (CV4) can significantly promote the expression of HLA-DR. After EA treatment, the expression of T lymphocyte subsets CD3+, and the CD4/CD8+ ratio significantly increased, thus regulating cellular immune function (43).

In summary, acupuncture therapy has a two-way benign adjustment effect on the immune system of patients with sepsis. In the stage of excessive immune response in patients with sepsis, acupuncture can achieve organ protection by inhibiting excessive activation of the immune system and the systemic inflammatory response. Acupuncture therapy can significantly improve immune function in patients with immunosuppressed sepsis. The duration of the acupuncture intervention is generally 20-60 min, and the treatment cycle is generally 3-10 days.

## 4 Animal models and acupuncture intervention parameters for the basic study of sepsis

### 4.1 Animal models of sepsis in the study of acupuncture mechanism

Currently, animal models used to study the mechanism of sepsis include the exogenous toxin model, exogenous live bacteria model, intraperitoneal infection model, and extraperitoneal sepsis model, with rats and rabbits as the main model animals. The exogenous toxin model involves systemic inflammation induced by intravenous or intraperitoneal injection of an endotoxin or LPS. The LPS model exhibits strong controllability and consistent animal reactivity. Exogenous live bacteria, such as *Escherichia coli*, can cause systemic inflammation through intraperitoneal or intravenous injection. The intraperitoneal infection model refers to a sepsis model that causes excessive inflammation through caecal ligation and puncture (CLP) (48) or colon ascendens stent peritonitis (CASP) (49). Necrotic tissue can be a source of inflammatory reactions. The CLP model (50) simulates a mixed bacterial infection caused by human appendicitis or diverticulitis. The characteristics of the CLP model are that the increase in cytokine levels is relatively gentle and lasts for a long time. The length of caecal ligation is a major factor affecting the mortality of the CLP model mice when the needle size and puncture times are controlled (51, 52). The CASP model involves the implantation of a stent with a fixed diameter into the ascending colon so that the intestinal contents continue to leak into the abdominal cavity, causing acute multi-bacterial sepsis peritonitis. The severity of sepsis can be adjusted by changing the stent diameter, or removing the stent during the second surgery and suturing the intestinal perforation (53, 54). The extraperitoneal sepsis model can induce lung infection by injecting bacteria into the trachea or nasal cavity, and can be used to study the local pathology of sepsis. This model is characterised by simple operation and high repeatability; however, its course is affected by the selected strains, dose, and antibiotics, which have certain limitations (55). The exogenous toxin and intraperitoneal infection models are commonly used to study the pathological mechanisms of sepsis. The CLP model was improved by Wichterman et al. and has been gradually standardised for more than 30 years (56, 57). As its pathological process is similar to the clinical symptoms, the CLP model is considered the gold standard for sepsis research (52, 58).

### 4.2 Rule of acupoint selection and acupuncture parameters in the study of acupuncture mechanism

We reviewed 54 basic studies on the acupuncture treatment of sepsis (**Table 2**) and found that 21 studies used ST36 alone as acupuncture intervention acupoints, three used ST36 and Feishu (BL13), three used ST36 and Neiguan (PC6), four used ST36 and Baihui (GV20/DU20), and six studies used ST36 combined with Tianshu (ST25), DU20, Quchi (LI11), Neiguan (PC6), and other acupoints. ST36 is the most commonly used acupuncture point in studies of sepsis mechanisms. Acupuncture at ST36 and BL13 improved sepsis-induced lung injury. Acupuncture with ST36 combined with GV20/DU20 improved brain injury in sepsis. In the fever and sepsis model (63, 90, 96, 99), acupoints Dazhui, LI11, and Yongquan were selected for acupuncture.

**Table 2:** Mechanism of acupuncture in preventing and treating sepsis.

Sepsis model	Species	Model induction method	Intervention methods	Acupoints	Parameter of acupuncture	Acupuncture Pretreatment or post-treatment	Test site Site + Mechanism	Refs.
Endotoxemia	Mice	LPS (ip)	MA, EA	ST36	MAC: 30 min; EAC: 1v, 1 Hz, 30 min	post-treatment	Plasma/spleen: TNF-α↓; DVC: c-Fos↑	Lim (2016) (59)
Endotoxemia	Mice	LPS (ip)	EA	ST36, ST25	10 Hz, 0-3.0 mA, 15 min	Pretreatment and post-treatment	Serum: TNF-α↓, IL-1β↓, IL-6↓; Serum catecholamine: NA↑, A↑, DA↑; spinal intermediolateral nuclei/suprarenal ganglia/DMV: c-Fos↑	Liu (2021) (60)
ALI	Rabbit	LPS (iv)	EA	ST36, BL13	2 Hz/15 Hz, ≤1 mA, 15 min	Pretreatment	Lung: W/D↓, HO-1↑, Nrf2↑, SOD↑, MDA↓; serum: CAT↑, GPx↑; Plasma: TNF-α↓, IL-6↓	Yu (2014) (61)
Endotoxic shock	Rabbit	LPS (iv)	EA	ST36, BL13	10 Hz, disperse-dense wave, 15 min	Pretreatment	Lung: W/D↓, HO-1↑, SOD↑, MDA↓, EB↓; Plasma: CO↑	Yu (2013) (62)
Fever	Rabbit	Bacterial endotoxin	MA	LI11	Lifting and propulsion; Amplitude: 2 mm, 60 cycles/min	post-treatment	serum: TNF-α↓, IL-1β↓, IL-4↑	Wang (2017) (63)
Myocardial Injury in Septic	Rat	CLP	EA	ST36	2-100 Hz, 2 mA, 1 hour	post-treatment	Plasma: CK-MB↓; cardiac muscle: TNF-α↓, NO↓, MPO↓	Zhang (2018) (64)
Endotoxemia	Rat	LPS (iv)	EA	ST36	2-100 Hz, 2 mA, 1.5 hour	post-treatment	Plasma: TNF-α↓, ALT↓, CK-MB↓, DAO↓, Cr↓	Song (2014) (65)
Sepsis	Mice	CLP	EA	ST36, GV20	2 Hz, 1 mA, 30 min, 2 times per day	post-treatment	Hippocampus: NO↑, p-eNOS↑, Aβ↓	Jun (2022) (66)
cognitive impairment	Mice	LPS (ip)	EA	GV20	2/100 Hz, 4 mA, dilatational wave	Pretreatment	Hippocampus: MDA↓, H2O2↓, GSH↑, CAT↑, IL-1β↓, IL-6↓, TNF-α↓, α7nAChR↑, ACh↑, ChAT↑, AChE↓	Han (2018) (67)
SAE	Mice	LPS (ip)	EA	ST36, DU20	1 mA, 30 min	post-treatment	Hippocampus: PICK1-TLR4↑, TLR4↓, p-ERK/JNK/P38↓; Plasma: IL-1β↓, IL-6↓, TNF-α↓	Mo (2021) (68)
Sepsis	Rat	CLP	EA	ST36, GV20	2-15 Hz, 2 mA, continuous wave, dilatational wave, intermittent wave, 5 days, 30 min	Pretreatment	serum and hippocampus: TNF-α↓, IL-6↓, MDA↓, SOD↑, CAT↑; hippocampus: TLR-4↓, NF-κB↓, Iba 1↓	Chen (2016) (69)
Endotoxemia	Rat	LPS (ip)	EA	LI4, PC6	2 Hz/100 Hz, 4 mA, 45 min	Pretreatment	Serum: TNF-α↓, IL-1β↓, IL-6↓	Song (2012) (70)
ABI	Rat	CLP	EA	ST36	2-100 Hz, 3 mA	post-treatment	Serum : TNF-α↓, HMGB1↓, ghrelin↑; bowel: MPO↓, DAO↑, GSH-R↑, ghrelin↑, HMGB1↓	Wu (2017) (71)
Sepsis	Rat	CLP	EA	ST36	2-100 Hz, 2 mA, 30 min	Pretreatment and post-treatment	Plasma: D-Lactose↓; Intestinal Mucosa: SIgA↑; CD3+T↑, γ/δ↑, CD4+ T↑, ratio of CD4+/CD8+ T cells↑	Zhu (2015) (72)
Hippocampal injury	Mice	LPS (ip)	EA	ST36, DU20	2/15 Hz, <1.5 mA, disperse-dense waves, 30 min	Pretreatment and post-treatment	Hippocampus: ROS↓, SOD↑, MDA↓, ATP↑	Mu (2022) (73)
ALI	Mice	LPS	EA	ST36	2 Hz, 1 mA, continuous wave, 10 min	Pretreatment	Serum/BALF: TNF-α↓, IL-1β↓, IL-6↓, IL-4↑, IL-10↑; lung: W/D↓, Sirt1↑, NF-κB↓, ac-NF-κB↓, ACE2↑	Luo (2022) (74)
SAE	Rat	CLP	EA	ST36, DU20, LI11	2 Hz/15 Hz, <1.5 mA, disperse-dense wave, 30 min	Pretreatment and post-treatment	Hippocampus: PSD-95↑, synaptophysin↑, MDA↓, SOD↑, Nrf2↑, HO-1↑	Li (2020) (75)
AKI	Rabbit	LPS (iv)	EA	ST36, PC6	2Hz/15 Hz, 1 mA, disperse-dense wave, 15 min	Pretreatment	Renal tissues: MDA↓, SOD↑, P-Akt↑, HO-1↑, Nrf2↑; Plasma : BUN↓, TNF-α↓, IL-10↑, Cr↓; Urine: NAG↓	Yu (2015) (76)
ALI/ARDS	Mice	LPS (ip)	EA	ST36	4/20 Hz, 0.5 mA, 20 min	post-treatment	Lung: W/D↓, MDA↓, GSH↑, ROS↓, GPX4↑, SLC7A11↑, FTH1↑, TNF-a↓, IL-1β↓, a7nAchR↑	Zhang (2022) (77)
Endotoxemia	Rat	LPS	EA	PC6	50 Hz, 1-3 mA(increased gradually), 4 v, 10 min	post-treatment	Plasma : ALT↓, AST↓, LDH↓	Liu (2011) (78)
Sepsis	Rat	CLP	EA	ST36	30 Hz, 40 mA, 20 min	post-treatment	Serum: TNF-a↓, IL-6↓, HMGB1↓, nitrite↓	Villegas (2014) (79)
AKI	Rat	LPS (iv)	EA	ST36, PC6	2 Hz, ≤1 mA, 30 min	Pretreatment	Renal tissue: iNOS↓, NF-kB↓; Plasma: TNF-a↓, IL-1β↓, IL-10↑, nitrite↓; BUN↓, Cr↓	Gu (2011) (80)
Sepsis	Rat	CLP	MA	ST36, GV01, B25, GV03, GV14, Liv02, Er Jien, LI11	/	post-treatment	Peritoneal lavage fluid: bacterial counts↓, neutrophil migration↑	Scognamillo (2004) (48)
Sepsis	Rat	CLP	EA	ST36	4/50 Hz, 3 mA, 30 min	post-treatment	Ileum: occludin↑; serum: D-lactate↓	Zhang (2018) (81)
Sepsis	Rat	CLP	EA	ST36, LI11, ST25	3 Hz, 2V, 15 min	post-treatment	Serum: TNF-α↓, IL-10↓, D-LA↓, DAO↓	Xie (2020) (82)
AKI	Rat	Eschericia coli ATCC (ip)	EA	ST36	2 Hz, 30 min	Pretreatment	Plasma: urea↓, creatinine↓	Harpin (2020) (84)
Injured lung induced by endotoxic shock	Rabbit	LPS (iv)	EA	ST36, BL13	2 or 100 Hz, disperse-dense wave, 30 min	Pretreatment	Lung: W/D↓, MDA↓, SOD↑, HO-1↑, p-ERK1/2↑; Serum: TNF-a↓	Zhang (2014) (83)
Endotoxemia	mice	LPS (ip); CLP	EA	ST36	40 mA, 4V, 15 min	Pretreatment and post-treatment	Serum: TNF-α↓, IL-6↓, MCP-1↓, INF-γ↓, DA↑, NE↑, E↑	Torres (2014) (85)
Sepsis	Rat	LPS (ip)	MA	ST36	twirled and twisted (>360°), 60 turns/min for 1 min, 30 min	Pretreatment and post-treatment	Plasma: BUN↓, Cr↓; kidney: PMN↓, MPO↓, iNOS↓, NO ↓	Huang (2007) (86)
ALI	Rat	LPS (ip)	MA	ST36	30 min	Pretreatment	Lung: NO↓, iNOS↓, MPO↓	Huang (2006) (87)
Sepsis	Mice	LPS (ip)	EA	ST36, ST25, BL56, LI10	10 Hz, 0.5/1.0/3.0 mA, 15 min	Pretreatment	Serum: TNF-α↓, IL-6↓, Catecholamine: NA↑, A↑, DA↑; DMV/vagal efferent neurons: Fos+↑	Liu (2021) (88)
Endotoxic shock	Rat	E.CoLi (iv)	EA	ST36, DU26	15 min	post-treatment	adrenal gland: SDH↑, ALP↑, Glycogen↑	Wang (1996) (89)
Fever	Rabbit	Escherichia coli endotoxin (iv)	MA, EA	GV14, LI11(left)	5 Hz, continuous bimodal pulse wave, 16 min	post-treatment	/	Kuang (1992) (90)
Endotoxin induced liver injury	Rat	LPS(iv)	EA	ST36	2-100 Hz, 2 mA, 1.5 hour	post-treatment	Liver: TNF-α↓; Plasma: ALT↓	Shi (2008) (91)
Endotoxemia	Rat	LPS (ip)	EA	BL32	30 Hz, 30 min	Pretreatment and post-treatment	Serum: TNF-α↓, IL-6↓, IL-1β↓	Wu (2021) (92)
Endotoxin shock	Rat	LPS (iv)	EA	PC6	2/14 Hz, 1 mA, Density wave	post-treatment	Plasma: TNF-α↓, NO↓	Li (2003) (93)
Endotoxin shock	Rat	E.CoLi (iv)	EA	ST36, DU26	15 min	post-treatment	Liver: G-6-Pase↑, SDH↑, 5´-Nase↑, Mg++-ATPase↑, liver glycogen↑	Huang (1995) (94)
ALI	Rat	CLP	EA	ST36, PC6	2-14 Hz, l mA, Density wave, 30 min	post-treatment	Lung: W/D↓, TNF-α↓, IL-6↓, P-JAK1↓, P-STAT3↓, Caspase-3↓, Bax↓	Xie (2020) (95)
Fever	Rabbit	ET (iv)	EA	KI1	8 Hz, 4.5 V/25 V, continuous square wave pulse, 4 cycles for 1 hour.	post-treatment	PO-AH: HSN↑	Dong (2008) (96)
Heat syndrome rabbits	Rabbit	ET	MA	LI11	/	post-treatment	Serum : ET↓; rectal temperature↓	Zhou (2012) (97)
Sepsis	Rat	CLP	EA	ST36	2/100 Hz, 2 mA, 1 hour	post-treatment	HBF↑; Plasma: ALT↓; liver: MDA↓, XOD↓	Shi (2010) (98)
Endotoxin induced fever model	Rabbit	ET (iv)	EA	CV14, LI11	12 Hz, 0.6-2 V, 10 min	post-treatment	Plasma/CSF: AVP↑; rectal temperature↓	Yang (1994) (99)
SE	Rat	CLP	EA	ST36	2-100 Hz, 2-3 mA, 30 min	post-treatment	Cerebral tissue: TNF-α↓, IL-6↓; Plasma: NSE↓	Wang (2013) (100)
Endotoxemia	Rat	LPS (iv)	EA	ST36, The auricular concha	10 Hz, 1 mA, 1 ms, 20 min	post-treatment	Serum: TNF-α↓, IL-6↓; Lung: NF-κB p65↓	Zhao (2011) (101)
Sepsis	Rat	CLP	EA	ST36	2-100 Hz, 2 mA, 30 min	post-treatment	Serum: HMGB1↓, Ghrelin↑; Jejunum tissue: HMGB1↓, Ghrelin↑	Wu (2014) (102)
ALI	Rat	LPS	MA	ST36	5 min, 4 days	Pretreatment	Blood/BALF: count of total leukocytes↓, count of total neutrophils↓	Ferreira (2009) (103)
Endotoxemia	Rat	LPS (ip)	EA	ST36	2 Hz, 1 mA, 5 min; 2 Hz, 1.5 mA, 5 min; 2 Hz, 2 mA, 20 min; continuous wave	Pretreatment	Serum: TNF-α↓, IL-1β↓, IL-6↓; Zusanli Acupoint/Serum: Ca^2+^↓; Spleen: CB2R↑, TLR4↓, NF-κB p65↓	Chen (2019) (104)
Peritonitis	Rat	LPS (ip)	MA	SP6	10 min	Pretreatment	Peritoneal fluid: MPO↓, TNF-α↓, IL-6↓, IL-10↑, leukocytes and neutrophil counts↓; brainstem: TNF-α↓, IL-6↓	Ramires (2021) (50)
Sepsis	Mice	LPS (ip)	EA	ST36	10 Hz, 0.1 mA, continuous wave, 30 min	Pretreatment	Serum: TNF-α↓, IL-1β↓, IL-5↓, IL-6↓, IL-10↓, IL-17A↓, eotaxin↓, IFN-γ↓, MIP-1β↓, KC↓	Lv (2022) (105)
Sepsis	Rat	CLP	EA	ST36	2 Hz, 2mA, 30 min	post-treatment	Small intestinal tissue: Bcl-2↑, Bax↓, IL-4↑; small intestinal mucus: sIgA↑	Lou (2022) (106)
septic cardiomyopathy model	Mice	LPS (ip)	EA	PC6, ST36	0.3 mA, 2 Hz, 30 min, once a day for 7 days	Pretreatment	Cardiac function: EF↑, FS↑, E/A↑; Serum and heart tissue: TNF-α↓, IL-1β↓; BAX\Bcl2↓; cardiomyocyte apoptosis↓; calpain-2↓; p-STAT3↓	Li (2022) (107)
Sepsis	Rat	LPS (ip)	EA	ST25	15 Hz, a pulse width of 1 ms, 3 mA, 20 min	post-treatment	Blood: NE↑, IL-10↑, IL-6↓, IL-1β↓	Zhang (2022) (108)
Sepsis	Rat	CLP	EA	PC6	2/15 Hz, 2 mA, 20 min	post-treatment	LF/HF↓, pH↑, BE↑, lactate levels↓, MAP↑; plasma: BNP↓, cTnI↓, TNF-α↓, IL-1β↓; cardiac tissue: α7nAChR↑; percent survival↑	Wu (2023) (109)
Endotoxemia	Mice	LPS (ip)	EA	ST36, LI4	2 Hz/15 Hz, 1 mA	Pretreatment	Intestine mucosa: ATP↑,DAO↑, ROS↓, OCR↓, HO-1↑, PINK1↑, Mfn1↑, Mfn2↑, OPA-1↑, Drp1↓, Fis1↓, caspase-1↓, IL-1β↓	Zhang (2023) (110)

↑, upregulated by acupuncture; ↓, downregulated by acupuncture.

LPS, lipopolysaccharide; ip, intraperitoneal Injections; MA, manual acupuncture; EA, electroacupuncture; ST36, Zusanli; MAC, manual acupuncture; EAC, electroacupuncture; TNF-α, tumor necrosis factor-alpha; DVC, the dorsal vagal complex; ST25, Tianshu; IL-1beta, interleukin-1beta; IL-6, interleukin 6; NA, noradrenaline; A,adrenaline; DA, dopamine; DMV, dorsal motor nucleus of the vagus nerve; IV,intravenous injection; BL13, Feishu; W/D, wet/dry; HO-1, heme oxygenase-1; Nrf2,nuclear factor erythroid-2 related factor-2; SOD, superoxide dismutase; MDA, malondialdehyde; CAT, catalase; ALI, acute lung injury; GPx, glutathione peroxidase; EB, evans blue; CK-MB, creatine kinase MB; NO, nitric oxide; MPO, myeloperoxidase; ALT, alanine aminotransferase; DAO, diamine oxidase; Cr, creatinine; GV20/DU20, Baihui; eNOS, endothelial nitric oxide; Aβ,β–peptide; H2O2, hydrogen peroxide; GSH, glutathione; α7nAChR, α7 nicotinic acetylcholine receptors; ACh, acetylcholine; ChAT, choline acetyltransferase; AChE, acetylcholinesterase;PICK1, protein Kinase C; TLR4, toll-like receptor 4; ERK1/2, extracellular signal regulated kinases1/2; JNK, the c-Jun N-terminal kinases; Iba 1, ionized calcium binding adaptor molecule 1; PC-6, Neiguan; HMGB1, high mobility group box-1; SAE, sepsis-associated encephalopathy; ABI, acute bowel injury; GSH-R, ghrelin receptor; ATP, adenosine triphosphate; BALF, bronchoalveolar lavage fluid; ACE2, angiotensin-converting enzyme 2; LI11, Quchi; PSD-95, postsynaptic density protein-95; AKI, acute kidney injury; BUN, blood urea nitrogen; Cr, creatinine; NAG, N-acetyl-glucosaminidase; ARDS, acute respiratory distress syndrome; ROS, reactive oxygen species; GPX4, glutathione peroxidase 4; SLC7A11, solute carrier family 7 member 11; FTH1, ferritin heavy chain 1; AST, aspartate aminotransferase; LDH, lactate dehydrogenase; iNOS, nitric oxide synthase; GV14, Dazhui; GV01, Changqiang; GV03, Yaoyangguan; D-LA, D-lactic acidosis; MCP-1, monocyte chemotactic protein-1; INF-γ, interferon-γ; NE, norepinephrine; E, epinephrine; DA, dopamine; PMN, polymorphonuclear neutrophil; NA, noradrenaline; DU26, Renzhong; SDH, succinate dehydrogenase; ALP, alkaline phosphatase; BL32, Ciliao; G-6-Pase, glucose-6-phosphatase; 5’ -Nase,5 ‘- Nucleotidase; NK, the c-Jun N-terminal kinases; STAT3, signal transducer and activator of transcription 3; Caspase-3, cysteine aspastic acid-specific protease 3; Bax, BCL-2-associated X protein; PO-AH, preoptic region and anterior hypothalamus; HSN, the heat sensitive neurons; ET, endotoxin; HBF, hepatic blood flow; CV14,juque; CSF, cerebrospinal fluid; AVP, arginine vasopressin; SE, septic encephalopathy; NSE, neuron-specific enolase; CB2R, cannabinoid CB2 receptor; SP6, sanyinjiao; KC, keratinocyte-derived chemokine; MIP-1β, macrophage inflammatory protein-1β; sIgA,secretory IgA; Bcl-2, B-cell lymphoma-2; EF, ejection fraction; FS, fractional shortening; E, A, early diastolic mitral annular velocity and late diastolic mitral annular velocity; CV6, Qihai; LF, low-frequency; HF, high frequency; pH, potential of hydrogen; BE, base excess; MAP, mean arterial pressure; BNP, brain natriuretic peptide; cTnI,cardiac troponin I; OCR, oxygen consumption rate; PINK1, PTEN-induced putative kinase 1; Mfn1/2, mitofusin 1 and 2; OPA-1, optic atrophy 1; Drp1, dynamin-related protein 1; Fis1, mitochondrial division protein 1.

In research on the mechanism of acupuncture treatment for sepsis, EA (45 studies) was the most commonly used acupuncture intervention. Generally, the duration of the EA treatment was 30 min, the depth of acupuncture was mostly 1.5–5 mm, and the range of stimulation intensity was mainly between 1 and 4 mA. Low-frequency (1, 2, and 10 Hz) and variable-frequency (2/14 Hz, 2/15 Hz, and 2/100 Hz) stimulations are commonly used. Strong and weak electrical stimulation can activate different anti-inflammatory signalling pathways. High-intensity (3 mA) EA at ST36 or ST25 activates neuropeptide Y (NPY) splenic sympathetic reflex through the spinal sympathetic axis to play an anti-inflammatory role. Low-intensity (0.5 mA) EA at ST36 can activate the vagus–adrenal axis to play an anti-inflammatory role, whereas ST25 low-intensity (0.5 mA) stimulation cannot activate this pathway (60). This shows a correlation between the intensity of EA intervention and its efficacy. In the comparison of waveforms, research has shown that dilation waves, intermittent waves, or continuous waves can inhibit the activation of microglia and reduce inflammation, oxidative stress, and apoptosis to reduce sepsis-induced brain damage; however, the effect of dilation waves is the most significant, followed by intermittent waves, while the phase contrast of continuous waves is poor (69, 99). This suggests that dilated wave preconditioning may be a promising therapeutic strategy for alleviating sepsis-induced brain injury. Additionally, before the sepsis model was established, high-intensity (3 mA) EA at ST36 or ST25 played an anti-inflammatory role by activating the NPY splenic sympathetic nerve reflex through the spinal sympathetic axis. The same intervention showed a proinflammatory effect after the sepsis model was established (60). This indicates that the pathological state of sepsis at the time of acupuncture affects the therapeutic effects of acupuncture.

In addition to EA, seven studies used manual acupuncture (MA) intervention in animal models of sepsis. Some studies have compared the efficacy of MA and EA and found that MA more effectively reduced the production of proinflammatory cytokines in the spleens of septic mice, whereas EA more effectively induced c-Fos expression than MA (59). However, whether this is associated with the parameters of the MA and EA interventions remains unclear. Therefore, the anti-inflammatory effects of EA and MA require further investigation. Acupuncture manipulation is closely related to curative effects. In the rabbit model of fever induced by endotoxin, it was found that ‘Shaoshanhuo’ (heat-producing needling) and ‘Toutianliang’ (cool-producing needling) both reduced rectal temperature and serum endotoxin levels, while the effect of a cold needle in reducing endotoxin content was relatively good (97).

## 5 Acupoint initiation mechanism of acupuncture for anti-inflammatory effects

Acupuncture stimulates the local acupoints to produce curative effects. Local mechanical stimulation at acupoints is converted into chemical signals that activate the body’s neuroendocrine-immune system and initiate the acupuncture effect (111, 112). The study found that collagen fibres (fascicular or reticular arrangement) in the LI11 acupoint may be the first material basis for the acupoint to perceive the acupuncture mechanical force. Acupuncture stimulation can cause deformation of collagen fibres in the extracellular matrix of the local acupoint and transmit mechanical signals to the surrounding connective tissue cells (63), producing cascade reactions. A research report published in *Nature* showed that the PROKR2 sensory neuron at the ST36 acupoint may be the anatomical basis for EA to drive the vagus adrenal axis in mice and play a role in inhibiting sepsis inflammation (88). These neurons control the deep fascia of the hind limb (such as the periosteum), which is crucial for driving the vagal-adrenal axis. In 2014, Ulloa et al. found that EA further activated aromatic l-amino acid decarboxylase to control systemic inflammation and improve the survival rate of septic mice by activating the sciatic nerve to induce the vagus nerve (85). Dong found that EA stimulation of acupoint KI1 antagonises the electrical activity of heat-sensitive neurons (HSN) in the preoptic area and hypothalamus induced by endotoxic heat, which may be caused by the activation of high-threshold thin nerve fibres at local acupoints (96). In addition, studies have shown that the change in Ca^2+^ influx in the ST36 acupoint area of endotoxic rats may serve as a bridge between local EA stimulation and systemic effects. Its potential mechanism may be that EA pretreatment reduces Ca^2+^ influx by activating the cannabinoid CB2 receptor (CB2R) at the ST36 local acupoint, thereby inhibiting the inflammatory response in endotoxic rats (104).

The above evidence shows that acupuncture may act on local fascicular or reticular collagen fibres through mechanical stimulation, causing local tissue deformation, further transmitting acupuncture signals to connective tissue cells, or activating sensory nerve fibres (PROKR2 neurons, high threshold thin nerve fibres, sciatic nerve) in the acupoint area, and at the same time, it may cause the release of bioactive chemicals (CB2R activation, Ca^2+^ influx) in the acupoint area, causing cascade reactions, thus playing a role in acupuncture.

## 6 Autonomic neurobiological mechanism of acupuncture in inhibiting sepsis inflammation

The interaction between the sympathetic and parasympathetic nerves of the autonomic nervous system and immune system affects inflammation. The vagus nerve is the main parasympathetic nerve responsible for the physiological regulation of most internal organs. The regulation by sympathetic nerves of the inflammatory response is affected by receptor subtypes expressed by neurotransmitters and immune cells, which have proinflammatory and anti-inflammatory effects. Both the sympathetic and parasympathetic nervous systems are involved in the acupuncture inhibition of systemic inflammation in sepsis, including the vagal cholinergic anti-inflammatory pathway, vagal adrenal medulla dopamine pathway, and sympathetic pathway.

### 6.1 Vagal cholinergic anti-inflammatory pathway

The cholinergic anti-inflammatory pathway (CAP) was first proposed by Borovikova et al. in the early 20th century (113). The vagus nerve CAP is a neuroimmune anti-inflammatory pathway that is mainly composed of nicotinic acetylcholine receptors (α7nAChR) containing α7 subunits on immune cells, and acetylcholine (ACh) released by the vagus nerve and its terminals. Compared with the humoral anti-inflammatory pathway, the cholinergic anti-inflammatory pathway has a very short reaction time. It rapidly and efficiently regulates systemic inflammatory responses and reduces mortality rates. Studies have shown that electroacupuncture can increase the activity of the vagus nerve, promote the expression of α7nAChR in macrophages in the myocardial tissue, prevent the occurrence of hyperlactatemia, alleviate the decline in left ventricular ejection fraction, inhibit the systemic and cardiac inflammatory response, and alleviate the histopathological manifestations of the heart (109). In the rat CLP model, EA ST36 can reduce the plasma activity of creatine kinase MB (CK-MB) and TNF-α in the myocardium and reduce the expression of nitric oxide (NO) and myeloperoxidase (MPO). When the bilateral abdominal vagus nerves are cut off, the effect of acupuncture on inhibiting the level of inflammatory factors in sepsis rats decreases significantly, suggesting that the cholinergic anti-inflammatory pathway is one of the main mechanisms of EA’s anti-inflammatory and myocardial protective effects (64). The auricular concha is distributed along the branches of the auricular vagus nerve. Both EA of the auricular concha and vagus nerve can increase serum TNF-α and IL-6 levels, and downregulate pulmonary NF-κB p65 expression levels in endotoxemia, with similar cholinergic anti-inflammatory mechanisms (101). Iron death has been shown to occur in alveolar epithelial cells of mice with sepsis. The inhibition of ferroptosis can reduce lung injury (114). Zhang et al. found that EA at the ST36 acupoint can activate α7nAchR on the surface of alveolar epithelial cells of lung tissue, inhibit LPS-induced iron death of alveolar epithelial cells, and reduce the lung inflammatory response (77). Parasympathetic branches of the sacral plexus near the Ciliao (BL32) acupoint. Studies have found that EA at BL32 can start the anti-inflammatory effect of the parasympathetic nervous system, improve the survival rate of rats with lethal endotoxaemia, and reduce the inflammatory cytokines TNF-α, IL-6, and IL-1β with its systemic anti-inflammatory effect. Pelvic nerve resection significantly reduces the effect of EA on BL32. The pelvic nerve-mediated parasympathetic pathway of the sacral plexus may have a more rapid effect than the cervical vagus nerve-mediated anti-inflammatory pathway (92). In addition, pretreatment of acupoint Hegu (LI4) with EA can significantly reduce the release of serum proinflammatory factors, such as TNF-α, IL-1β, and IL-6; therefore, it can reduce the systemic inflammatory reaction, significantly improve the survival rate of rats with fatal endotoxemia, and EA at LI 4 has a stronger protective effect than at PC6 acupoint, while electrical stimulation of non-acupoints is ineffective. The protective effects of EA can be eliminated when the vagus nerve is cut off, the spleen is excised, or the central muscarine receptor and surrounding nicotine receptors are inhibited (70). Lim et al. found that acupuncture signals were transmitted to the dorsal vagal complex (DVC) and activated the splenic nerve through vagal activity, inducing an anti-inflammatory response in splenic macrophages (59). Organ failure due to excessive inflammation is the main cause of early mortality in patients with acute pancreatitis (AP). Zhang confirmed through experiments that EA can inhibit the infiltration of macrophages in the pancreas, reduce plasma amylase and TNF-α, IL-1β, and IL-6 expression, and inhibit systemic inflammation, and that cervical vagotomy or blocking α7nAChR can inhibit the protective effect of EA on the pancreas (115). The above research shows that the vagus cholinergic anti-inflammatory pathway can regulate the immune response and production of proinflammatory cytokines, which is an important acupuncture anti-inflammatory mechanism.

### 6.2 Vagus adrenal medulla dopamine pathway

In 2014, Ulloa et al. found that in mice with sepsis, EA increased the serum levels of three catecholamines, mainly dopamine and norepinephrine, and inhibited serum TNF, MCP-1, IL-6, and INF through the sciatic nerve activating the vagus adrenal medulla dopamine pathway-γ; therefore, reducing the systemic inflammatory reaction can improve the survival rate. EA with a wooden stopcock or stimulation with a non-acupuncture acupoint did not inhibit inflammatory levels. Capsaicin agonists, reserpine, sciatic nerve or neck, subphrenic vagotomy, and adrenalectomy can eliminate the anti-inflammatory effects of EA (85). In a rat model of CLP-induced sepsis, the effect of EA on reducing the levels of serum inflammatory factors depended on the integrity of the vagus nerve and catecholamine production (79). These studies have shown that EA can inhibit the systemic inflammatory reaction in mice with sepsis and improve the survival rate by activating the vagus nerve-adrenal medulla dopamine pathway through the sciatic nerve. In 2020, Professor Ma Qiufu of Harvard Medical School found that EA stimulation drives sympathetic pathways in somatotopy- and intensity-dependent manners. Low-intensity EA stimulation of ST36 (0.5 mA, 10 Hz) can activate the vagus adrenal axis anti-inflammatory pathway; however, low-intensity EA stimulation of ST25 does not affect the vagus nervous system or sympathetic nerves. In 2021, it further revealed the specific neuroanatomical basis of EA stimulation and found that low-intensity EA stimulation of ST36 acupoints in mice drove the vagal-adrenal anti-inflammatory axis to achieve an anti-inflammatory effect mediated by neurons expressing Prokr2 at the ST36 acupoint (88). Therefore, low-intensity EA stimulation may inhibit inflammation by activating the vagus nerve of the parasympathetic nervous system.

### 6.3 Sympathetic nerves

The sympathetic nervous system plays a dual role in regulating inflammatory responses and mediates both proinflammatory and anti-inflammatory effects. A previous study found that stimulating the homosegmental acupoint ST25 with 3 mA EA can drive the release of peripheral NE from the sympathoadrenal medullary axis (108). Unlike low-intensity EA, high-intensity EA activates the sympathetic nervous system and inhibits sepsis-induced inflammation. Before LPS-induced systemic inflammation, 3 mA high-intensity EA stimulation of ST25 on the abdomen can activate peripheral NPY+sympathetic neurons projecting to immune organs such as the spleen and play a role in β2-norepinephrine receptor-mediated anti-inflammatory effects. However, after LPS-induced inflammation, the application of the same abdominal acupoints and stimulation intensity showed obvious proinflammatory effects, mainly because LPS can induce and promote inflammation and increase the expression of α2-adrenergic receptors. High-intensity EA at ST36 can activate the ‘spinal sympathetic’ reflex, thus playing an anti-inflammatory role. Similar to the ST25 acupoint, high-intensity EA stimulation of the ST36 acupoint after LPS-induced inflammation has an inflammatory effect (60). This shows that high-intensity EA stimulation (3 mA) of ST36 or abdominal ST25 acupoints can activate the spinal cord and peripheral sympathetic reflexes, respectively. Moreover, the inflammatory regulatory effect of EA stimulation is bidirectional and related to the inflammatory state of the body during the acupuncture intervention.

The above results reveal that acupuncture at body surface acupoints can induce multiple somatosensory autonomic nerve target organ reflex pathways and regulate immune inflammation. This regulatory effect is related to acupoint location, stimulus intensity, and body state and has the characteristics of acupoint specificity, intensity dependence, and bidirectionality. These studies enrich the modern scientific connotation of body surface therapies, such as acupuncture and moxibustion, and provide an important scientific basis for the clinical optimisation of acupuncture parameters, inducing different autonomic nerve reflexes, and thus treating specific diseases (such as sepsis).

## 7 Acupuncture prevents sepsis organ dysfunction by inhibiting inflammation, antioxidative stress and reducing apoptosis

The regulation of acupuncture in the body is characterised by multiple systems, pathways, and targets. The response of the target organs is the key link for acupuncture to take effect. Acupuncture regulates the nerve endocrine immune system (NEI) of the body through the activation of the ‘acupoint network’, through integrating nerve conduction, and has a therapeutic effect through the ‘effect network’. Multiple-organ dysfunction syndrome (MODS) is the primary cause of death in sepsis. In sepsis, excessive inflammation, oxidative stress, and apoptosis are the main mechanisms that lead to multiple organ dysfunction. Acupuncture can reduce the levels of proinflammatory cytokines, increase the levels of anti-inflammatory cytokines, regulate the balance of prinflammatory and anti-inflammatory factors in two directions, reduce oxidative stress, improve cell energy metabolism, maintain mitochondrial function, and regulate cell apoptosis to prevent the progressive aggravation of sepsis, leading to MODS.

### 7.1 Lung

The lungs are the earliest organs damaged in sepsis with MODS (116–118). Acute lung injury (ALI) is an independent risk factor for organ dysfunction and death in sepsis patients (119). Acupuncture has a significant effect on relieving acute lung injury (103). During the development of ALI induced by endotoxins, the content of the oxidative stress products malondialdehyde (MDA), myeloperoxidase (MPO), and NO increased significantly, while that of superoxide dismutase (SOD) decreased significantly, leading to increased apoptosis (120). Heme oxygenase-1 (HO-1) is a rate-limiting enzyme in the process of heme catabolism and is a strong negative regulator of oxidative stress in the endotoxins of ALI and pneumonia. Its metabolites, bilirubin, carbon monoxide, and iron have the effects of antioxidative damage (121). Several studies have shown that HO-1 mediates the ability of acupuncture to relieve sepsis-induced lung injury. EA pretreatment at ST36 and BL13 acupoints for 5 days significantly increased the plasma CO content, reduced MDA and MPO, the number of apoptotic cells in the lung, and wet/dry (W/D) lung weight ratio, and reduced ALI caused by endotoxic shock, whereas acupuncture at non-acupoints showed no significant effect. Further research showed that EA preconditioning for 5 days promoted the expression of HO-1 mRNA and protein levels in ALI lung tissue, and the protective effect of EA preconditioning on septic lung injury disappeared after tail vein injection of the HO-1 inhibitor ZnPP IX, indicating that acupuncture protects the lungs by promoting the expression of HO-1 (62). In another report, acupuncture at ST36 significantly reduced the expression of lung-induced nitric oxide synthase (iNOS) and NO biosynthesis, and reduced LPS-induced acute lung injury in rats (87). Nuclear factor erythroid-2 related factor-2 (Nrf2) is a cap n-collar alkaline leucine zipper transcription factor that promotes the transcription of HO-1 by combining with the antioxidant response element (ARE). The Nrf-2/HO-1 signalling pathway is considered an antioxidant and protective pathway, especially in inflammatory diseases, including sepsis (122). The study found that pretreatment of bilateral ST36 and BL13 acupoints with EA promotes the expression of Nrf2 and HO-1 in ALI rabbit lung tissue and reduces the inflammatory factors TNF-α and IL-6 (61). This suggests that the promotion of HO-1 expression by EA pre-conditioning may be related to the Nrf2/ARE signalling pathway. In addition, Zhang found that EA stimulation of ST36 and BL13 upregulates HO-1 in the lungs of endotoxic shock rabbits, and the potential mechanism of reducing lung injury may be related to the upregulation of signal transduction of the extracellular signal-regulated kinases1/2 (ERK1/2) pathway (83). NAD-dependent deacetylase sirtuin 1 (SIRT1) can inhibit NF-κB signal activation, reducing oxidative stress and apoptosis, which is important role organ protection (123). Angiotensin-converting enzyme 2 (ACE2) is a single carboxyl peptidase that is important in maintaining the renin-angiotensin system (RAS). RAS can weaken immunity, inflammation, and other physiological activities (124), and ACE2 can prevent inflammatory damage in alveolar type II (AT II) cells by activating SIRT1-related pathways (125). The study found that EA pretreatment of ST36 acupoint for 7 days can more effectively improve LPS-induced acute lung injury than for 1 day, and promote the expression of ACE2 and SIRT1 in lung tissue, thereby reducing the acetylation modification of NF-κB. Reducing the inflammatory cytokines TNF-α, IL-1β, and IL-6 alleviated ALI (74). The JAK1/STAT3 signalling pathway is one of the key pathways that inhibits the polarisation of M2 macrophages with an inflammatory response (126). EA at ST36 can inhibit the release of TNF-α and IL-6, reduce apoptosis, and reduce lung injury in septic rats by activating the JAK1/STAT3 pathway (95). Therefore, acupuncture may alleviate acute lung injury caused by sepsis by inhibiting oxidative stress and inflammatory reactions. In this process, HO-1 is the key molecule for acupuncture to inhibit oxidative stress, SIRT1/NF-κB and JAK1/STAT3 signal pathways are involved in the process of acupuncture inhibiting inflammatory reaction.

### 7.2 Brain

Sepsis-associated encephalopathy (SAE) is a common complication of severe sepsis and is associated with high mortality rates in the intensive care unit. Generally, sepsis survivors have obvious cognitive deficits that may lead to poor quality of life (127). The degree of hippocampal injury induced by inflammatory reactions and oxidative stress positively correlates with cognitive dysfunction in sepsis (128). Several studies have shown that acupuncture alleviates sepsis-induced cognitive dysfunction by inhibiting neuroinflammation and oxidative stress.

ACh is a neurotransmitter necessary for maintaining normal cognition and memory, and its expression is positively correlated with cognitive ability (129). Choline acetyltransferase (ChAT) catalyzes ACh synthesis, while acetylcholinesterase (AChE) catalyzes ACh hydrolysis and degradation (130). In the SAE mouse model, EA significantly reduces AChE activity in the hippocampus, increases ChAT activity and ACh content, and enhances α7nAChR protein expression, reducing IL-1β, IL-6, and TNF-α levels to reduce neuroinflammation. This indicates that acupuncture can inhibit inflammation in the hippocampus of SAE mice through a cholinergic anti-inflammatory pathway (67). PICK1 is an important regulatory protein involved in brain-related diseases and plays a protective role in sepsis. PICK1 can bind to the TLR4 receptor on microglia and remain in the cytoplasm, preventing TLR4 from reaching the cell membrane and mediating inflammation. Mo et al. found that EA significantly increased the formation of the PICK1-TLR4 complex, thereby inhibiting the expression of proinflammatory cytokines (68). Neuron-specific enolase (NSE) is a clinical index used to assess the degree of central nervous system injury and prognosis. In the rat model of CLP-induced sepsis, EA at ST36 significantly reduced the plasma NSE level and the expression of proinflammatory cytokines in brain tissue, such as TNF-α and IL-6, reducing brain damage (100).

Endothelial nitric oxide (eNOS) is a calcium-dependent protease that exists in vascular endothelial cells and can synthesise trace amounts of NO, keeping the endothelium smooth and intact, maintaining vascular tension, and preventing thrombosis. β-peptide (Aβ) is formed by β-Amyloid precursor protein produced and released into the extracellular space. Under normal conditions, Aβ is swallowed up by microglia in the brain. However, when the Aβ concentration is too high, large plaques will form that damage neurons, and they will also enter the synaptic space to prevent memory formation. p-eNOS reduction and Aβ deposition are closely related to long-term cognitive deficits. Previous studies have shown that EA intervention can prevent and treat long-term cognitive dysfunction after sepsis-induced brain injury. The ST36 and GV20/DU20 acupoints are the most effective combination for improving cognitive function (131). The latest research confirms that EA GV20 and ST36 acupoints can increase the level of NO and p-eNOS and reduce Aβ in the hippocampus of sepsis-surviving mice and improve the long-term cognitive impairment caused by sepsis. However, intraperitoneal injection of the eNOS inhibitor L-NAME weakened the efficacy of EA therapy (66). Improving mitochondrial dysfunction is the key to preventing oxidative stress in the brain during sepsis. HO-1 is an endogenous protective substance essential for maintaining mitochondrial function (132). Mu et al. found that EA pretreatment of acupoints DU20 and ST36 significantly increased the activities of mitochondrial respiratory chain complexes I, II, III, and IV, as well as the levels of SOD and ATP, and reduced the levels of the oxidative stress products, ROS and MDA, in the hippocampi of septic mice. This suggests that EA prevents LPS-induced oxidative damage and mitochondrial respiratory defects. Interestingly, HO-1 knockout increases ROS and MDA levels and decreases SOD and ATP levels. HO-1 deficiency aggravates mitochondrial swelling, crest relaxation, and vacuole degeneration, indicating the key role of HO-1 in maintaining mitochondrial energy production and resisting oxidative damage in the hippocampus (73). In an SAE rat model, Li et al. found that EA DU20, ST36, and LI11 acupoints upregulated the expression of Nrf-2 mRNA and HO-1 protein, increased the SOD level, reduced the MDA level in the hippocampus, reduced the loss of neurons in the hippocampal CA1 area, and increased the 14-day survival rate (75). In addition, acupuncture reduced the levels of MAD and hydrogen peroxide (H2O2), increased the levels of catalase (CAT) and glutathione (GSH) in the hippocampus, and inhibited oxidative stress (67). This suggests that EA may exert antioxidant and protective effects against SAE by activating the Nrf-2/HO-1 pathway.

Acupuncture may increase PICK1-TLR4 complex formation, reduce plasma NSE levels and inhibit inflammatory reactions through the acetylcholine pathway, protect mitochondrial function by activating the Nrf-2/HO-1 pathway, increase p-eNOS, and reduce Aβ deposition, thus alleviating hippocampus damage during sepsis.

### 7.3 Intestines

Systemic hyperinflammation can lead to intestinal ischaemia, oedema, increased intestinal mucosal permeability, and translocation of intestinal bacteria and toxins, subsequently causing heterogeneous sepsis and MODS. It is important to effectively inhibit the production of proinflammatory factors in the early stages and protect the intestinal mucosal barrier for the prevention and treatment of sepsis.

Studies have shown that acupuncture significantly improves sepsis-associated intestinal hyperinflammation. Ghrelin is a brain-gut peptide secreted by endocrine cells of the gastrointestinal tract, which can inhibit the proinflammatory factors HMGB1 and TNF-α. Wu et al. found that the ST36 acupoint in EA sepsis model rats increased the expression rate of ghrelin-immunopositive cells in the intestine and reduced the serum and intestinal content of HMGB1. Correlation analysis revealed that HMGB1 levels decreased with increased ghrelin expression. However, ghrelin receptor blockers can block the inhibition of the inflammatory mediator HMGB1, proving that ghrelin mediates EA to inhibit intestinal inflammation (71, 102). HO-1 catalyses the breakdown of heme into free iron, carbon monoxide, and biliverdin, thereby regulating mitochondrial homeostasis and preventing oxidative cell damage (133, 134). TEN-induced putative kinase 1 (PINK1), the only kinase located mainly in the mitochondrial intima, has been found to exert a protective effect on the mitochondria during cellular stress. Studies have shown that EA induces the translocation of HO-1 to the mitochondrial inner membrane; activates PINK1; increases ATP production, DAO activity, and OCR; regulates mitochondrial fusion/division balance; reduces ROS content and OCR; and protects intestinal barrier function (110).

Increased intestinal mucosal permeability is a pathological mechanism underlying heterogeneous sepsis. The intestinal tight junction (TJ) barrier is an important component of the intestinal mucosal barrier. Intestinal epithelial cell tight junction (occludin) is an important protein in the intestinal epithelial cell TJ, and is negatively related to the permeability of the cell gap (135, 136). Monitoring blood D-lactate levels can also reflect changes in intestinal permeability and the degree of damage (137). Zhang et al. found that EA stimulation of ST36 increased the expression of occludin in rats with sepsis, reduced the level of serum D-lactate, and maintained the intestinal mucosal barrier (81). The immune barrier, composed of lymphocytes and secretory immunoglobulin A (sIgA) from humoral immunity, is crucial for maintaining the intestinal mucosal immune function and inhibiting intestinal bacterial translocation (138, 139). In the blood of septic mice, the concentration of D-lactose, a biomarker of intestinal permeability produced by intestinal bacteria, increased significantly (140). Zhu et al. found that EA pretreatment of ST36 significantly reduced the level of serum D-lactose, increase the concentration of sIgA in the intestinal mucosa, increase CD3+, γ/δ, CD4+T cell percentage, and CD4+/CD8+T cell ratio, reduce the permeability of CLP rats, and improve intestinal injury (72). Xie et al. also found that EA at ST36 and LI11 can restore intestinal T cell immune function to normal and significantly reduce TNF-α and IL-10 in the serum of CLP model rats. When the spleen is removed, EA ST36 cannot reduce blood TNF-α, IL-10, and D-LA levels; however, it can improve intestinal immune function by balancing the proportion of T lymphocytes (CD3+CD4+/CD3+CD8+cells and Treg/Th17 cells). Therefore, the spleen may be necessary for EA at ST36 to improve systemic inflammation, but regulation of the intestinal barrier and immune defence is not essential (82).

The above studies show that EA can inhibit intestinal hyperinflammation in sepsis, protect the intestinal mucosal barrier by regulating the immune function of intestinal lymphocytes, prevent intestinal bacterial translocation, and improve intestinal injury.

### 7.4 Kidney

Sepsis-associated acute kidney injury (SAKI) seriously affects the prognosis of patients with sepsis and increases mortality (141). EA has a significant inhibitory effect on oxidative stress and excessive inflammatory reactions in patients with SAKI. For example, Harpin et al. found that EA pretreatment at the ST36 acupoint significantly reduced urea and creatinine levels in SAKI rat models and protected renal function (84). Endotoxic shock results in an abnormal adrenal glucose metabolism and decreased adrenal glycogen levels. Succinate dehydrogenase (SDH), located in the mitochondria, is a key enzyme involved in the oxidative phosphorylation of glucose metabolism. The main function of alkaline phosphatase (ALP) is to increase phosphate metabolism, plasma membrane permeability, and the transport function of cells (142). In a rat model of endotoxic shock, EA at Renzhong (DU26) or ST36 prevented the loss of glycogen in the adrenal gland, enhanced the activities of SDH and ALP, and enhanced adrenal cortex function in endotoxic shock rats (89). The phosphoinositide 3-kinase (PI3K)/Akt signalling pathway controls the activation of Nrf2 and participates in the induction of HO-1 (143–145). In a rabbit model of acute renal injury, EA stimulation of the ST36 and PC6 acupoints significantly inhibited the LPS-induced increase in the AKI biochemical indicators, blood urea nitrogen (BUN), creatinine (Cr), and N-acetyl-glucosaminidase (NAG). EA at these acupoints also increases the PI3K/Akt/Nrf2 pathway and HO-1 protein expression, increases SOD and IL-10 levels, reduces MDA and TNF-α levels, and reduces apoptosis of renal tubular cells, thereby reducing renal injury. Intravenous injection of wortmannin, a PI3K/Akt pathway inhibitor, weakened the partial protective effect of EA. This indicated that the PI3K/Akt/Nrf2/HO-1 pathway mediates the protective effects of acupuncture (76). During sepsis, overactivation of the NF-κB signalling pathway induced the expression of iNOS and excessive production of NO level, which affected renal hemodynamics (146–149). Research has shown that acupuncture pretreatment at ST36 significantly reduces the overexpression of iNOS and the resulting NO in SAKI rats. However, acupuncture after sepsis onset had no obvious protective effects against renal injury (86). Another report demonstrated that EA at ST36 and PC6 acupoints inhibits NF-κB activity in renal tissue of SAKI rats, reduces iNOS expression in the kidney and plasma BUN and Cr levels, inhibits the release of TNF-α and IL-1β, and increases the release of anti-inflammatory factor IL-10 to reduce renal injury induced by endotoxaemia (80). These studies indicate that EA may pass through the PI3K/Akt/Nrf2/HO-1 and NF-κB signalling pathways and inhibit renal hyperinflammation and oxidative stress.

### 7.5 Liver

Liver injury is a manifestation of sepsis and multiple organ dysfunction syndrome. Liver injury can occur at all stages of sepsis and is an independent risk factor for MODS and death. An increasing number of studies have confirmed that early acupuncture intervention can improve the prognosis of patients with sepsis and liver injury. Various liver enzymes, including alanine aminotransferase (ALT), aspartate aminotransferase (AST), and lactate dehydrogenase (LDH), enter the blood circulation after liver cell injury in sepsis, and their activities can reflect the degree of liver injury. EA at PC6 significantly reduced the biochemical indicators ALT, AST, and LDH in rats with septic liver injury and reduced the deterioration of liver dysfunction in rats with endotoxic shock (78). In addition to enzyme system disorders, endotoxic shock is often accompanied by abnormal glucose metabolism and impaired cell membrane transport. In the early stages of shock, hyperglycaemia commonly causes hypoglycaemia as the shock deepens and blood glucose drops. Glucose-6-phosphatase (G-6-Pase) is a marker enzyme in the endoplasmic reticulum and a key enzyme in glucose metabolism. Glucose 6-phosphate is hydrolyzed into glucose into blood circulation through G-6-Pase, which is an indicator of the degree of liver cell damage and recovery; 5′-Nucleotidase (5′-Nase) and magnesium-activated adenosine triphosphate (Mg++- ATPase) are plasma membrane marker enzymes related to membrane transport function and also reflect the early damage of liver cells. Research has shown that EA at acupoints DU26 and ST36 increases the expression of G-6-Pase, SDH, 5′-Nase, and Mg++-ATPase, improve glucose metabolism, increase energy production, activate liver cell function, and improve the transport function of the liver cell membrane in septic rats (94). TNF-α is a multifunctional proinflammatory cytokine that induces an inflammatory storm, and most of it is synthesised in the liver, leading to sepsis and liver damage. Studies show that EA at ST36 can reduce TNF-α content in the liver tissue of rats with endotoxin-induced liver injury and plasma ALT activity to protect liver tissue (91). During sepsis, the liver is prone to ischaemia-reperfusion and oxygen-free radical damage. Increasing the blood flow to the liver tissue reduces tissue lipid peroxidation and oedema and improving liver damage. In a rat model of sepsis, EA ST36 reduced the MDA content in liver tissue, the activity of xanthine oxidase (XOD), and the expression of plasma ALT, improved tissue dysfunction, reduced liver oedema, and increased liver blood flow to improve ischaemia (98).

### 7.6 Heart

Myocardial injury is a common complication of septic shock and is an important reason for the poor prognosis of sepsis. Effective prevention and treatment of myocardial injury are important aspects of sepsis treatment. STAT3, a protein that promotes survival and inflammation in the heart, is closely associated with calpain in cardiomyocytes. Studies have shown that EA pretreatment can downregulate the expression of calpain-2, thereby inhibiting STAT3 phosphorylation and improving cardiac inflammation and dysfunction (107). In sepsis or MODS, NO loses control and aggravates organ damage. NO is an endothelium-derived vasodilator that relaxes the blood vessels. NO is a substrate of MPO. MPO is a peroxidase secreted by neutrophils, monocytes, and macrophages, and is involved in myocardial reperfusion injury. When myocardial ischaemia/reperfusion occurs, neutrophil infiltration can produce a large number of lysosomal enzymes, such as MPO, which not only reflects the number of neutrophils infiltrating the myocardium, but also the degree of activation. It can be used as a marker of neutrophil infiltration during myocardial injury. MPO reduces NO bioavailability and inhibits NOS activity. iNOS is expressed only after cells are stimulated by lipopolysaccharides, cytokines, and other factors. Lipopolysaccharide-activated iNOS can produce NO and inhibit myocardial contractility, and myocardial injury can be accompanied by an increase in the levels of the myocardial enzyme CK-MB. In a rat CLP model, EA reduced the levels of CK-MB in the plasma and TNF-α in the myocardium, and the activity of NO and MPO reduced the oedema of heart tissue and protected the myocardium (64). Song also found that EA at the ST36 acupoint reduced CK-MB and TNF in plasma-α horizontal when the α7 subunit of cholinergic N receptor was antagonised by α-BGT. Bilateral cervical vagotomy can also aggravate organ dysfunction and weaken the protective effect of EA on the myocardium. In addition, EA can reduce the levels of ALT, Cr, and DAO in the plasma, indicating that EA has protective effects in multiple organs in endotoxemic rats (65).

The above research shows that EA has a protective effect on the lungs, brain, intestines, kidneys, liver, and heart. Acupuncture can resist oxidative stress, inhibit excessive inflammation, improve cell energy metabolism, maintain mitochondrial function, regulate cell apoptosis, prevent multiple organ damage, and maintain and improve the integrity of body tissues, cell morphology, and function. In addition, EA significantly reduced the T lymphocyte apoptosis or pyroptosis in septic mice (105, 106). Electro-acupuncture expresses an anti-endotoxin shock effect by repressing the plasmic NO and TNFalpha concentrations smoothly and retrieving the blood pressure stably (93). In future medical research, it will be necessary to carry out multi-level comprehensive exploration at the whole-body, organ, cell, molecule, gene, and other levels, accurately understand the key links and regulatory pathways of network effects between major cells or inflammatory mediators, and deeply understand and clarify the pathogenesis of sepsis to effectively improve the treatment of sepsis and MODS.

## 8 Conclusion

**Figure 2:**
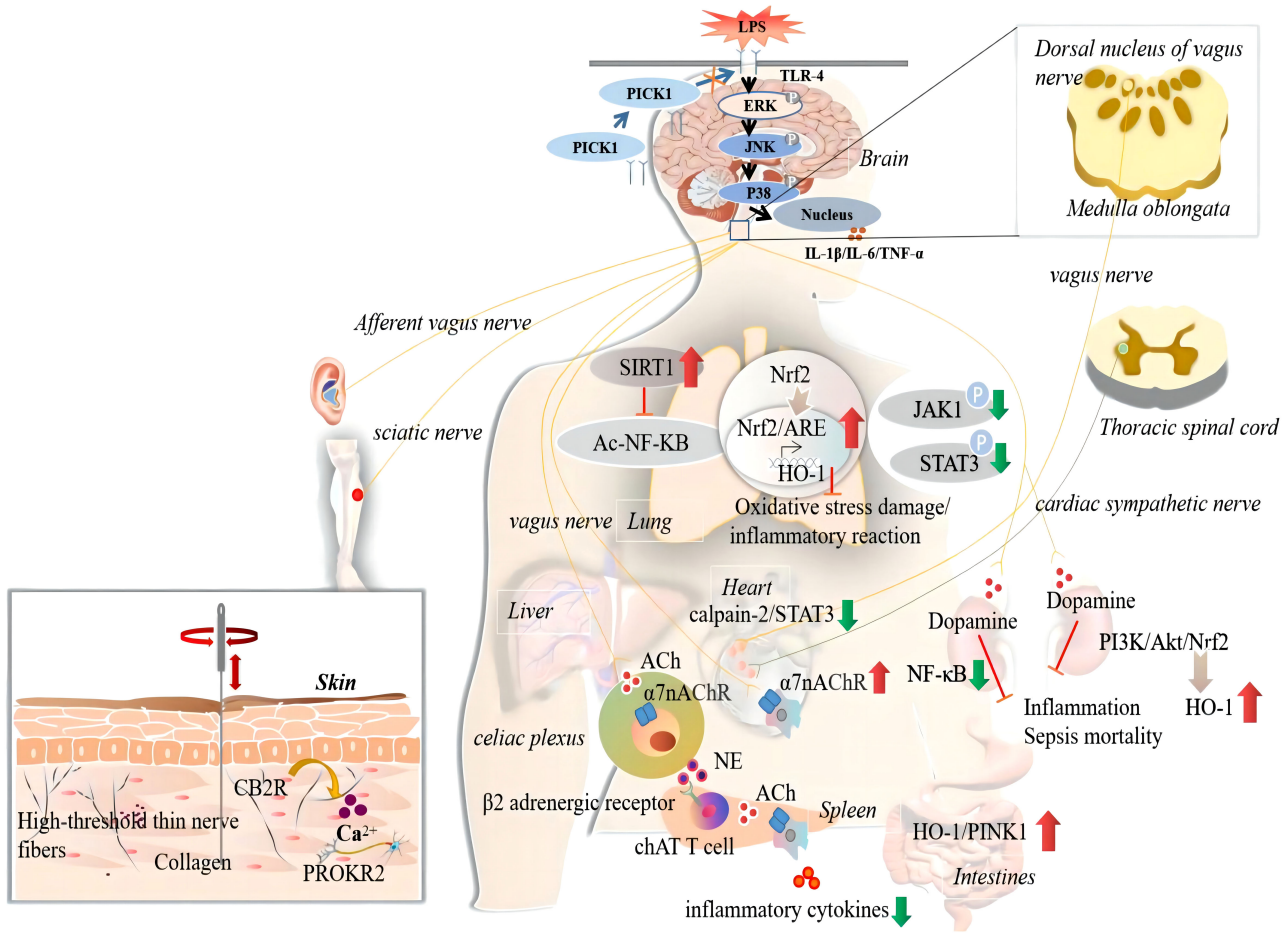
The anti-inflammatory actions and mechanisms of acupuncture in sepsis. The ‘↑’ represents upregulated by acupuncture; The ‘↓’ represents downregulated by acupuncture. CB2R, cannabinoid CB2 receptor; PICK1, protein Kinase C; ERK, extracellular signal-regulated kinase; JNK, the c-Jun N-terminal kinases; IL-1beta, interleukin-1beta; IL-6, interleukin 6; TNF-α, tumor necrosis factor-alpha; SIRT1, sirtuin 1; Ac, acetylation; NF-kB, the nuclear factor-kappa B; TLR4, toll-like receptor 4; Nrf2, nuclear factor erythroid-2 related factor-2; ARE, antioxidant response element; HO-1, heme oxygenase-1; JAK1, janus kinase 1; STAT3, signal transducer and activator of transcription 3; ACh, acetylcholine; α7nAChR, α7 nicotinic acetylcholine receptors; NE, norepinephrine; ChAT, choline acetyltransferase; P13K, phosphatidylinositol 3 kinase; Akt, protein kinase B; PINK1, PTEN-induced putative kinase 1.

The degree of imbalance in immune homeostasis is the core event in the disease process and determines the severity of sepsis. We systematically reviewed the animal model and acupuncture intervention parameters for the basic study of sepsis and the key molecular mechanisms underlying its anti-inflammatory effects (**Table 2**). In clinical studies, acupuncture has been shown to bidirectionally regulate the body’s immune system, inhibit systemic inflammatory responses, and improve the immunosuppressive state of patients with sepsis to improve or delay the pathological process of sepsis. In preclinical studies, exogenous toxin models, represented by intraperitoneal or intravenous injections of lipopolysaccharide (LPS), and intraperitoneal infection models of CLP are commonly used to study the pathological mechanism of sepsis. Acupuncture intervention is usually performed using a single acupoint or a multi-acupoint combination, among which ST36 is the most commonly used. The intervention methods and parameters of acupuncture, such as MA, EA, intensity, duration, depth, and waveform, are closely related to its effects. At a local acupoint, the initial mechanism of action of acupuncture is closely related to the release of collagen fibres and a number of biochemical substances in the acupoint microenvironment. PROKR2 neurons, high-threshold thin nerve fibres, CB2R activation, and Ca^2+^ influx are the key material bases for the transmission of acupuncture information from the acupoint area to the central nervous system. Different stages of acupuncture intervention can produce opposite effects. High-intensity EA at ST36 and ST25 had anti-inflammatory effects if applied before modelling, but promoted inflammatory responses if applied after modelling. Acupuncture can exert anti-inflammatory effects through the vagus-adrenal medullary-dopamine, vagus-adrenal axis/spinal sympathetic, vagus-spleen cholinergic, or transcutaneous auricular vagus-cholinergic anti-inflammatory pathways. Acupuncture at acupoints on the body surface can induce various somatosensory-autonomic nervous target organ reflex pathways that play a role in the regulation of immune inflammation in the body. However, this regulatory effect of acupuncture is related to the location of acupoints, stimulation intensity, and body state, and has the characteristics of acupoint specificity, intensity dependence, and bidirectionality. Specifically, low-intensity EA at ST36 can activate the vagus-adrenal axis or vagus-cholinergic pathway of the parasympathetic nervous system to inhibit systemic inflammation in sepsis. Acupuncture intervention before and after modelling has an anti-inflammatory effect, but low-intensity EA at ST25 has no anti-inflammatory effect. High-intensity EA stimulation can activate the spinal cord or the peripheral sympathetic nervous system to play an anti-inflammatory role. High-intensity EA at ST36 can activate the spinal sympathetic reflex to inhibit systemic inflammation in sepsis, whereas high-intensity EA at ST25 can activate NPY peripheral sympathetic neurons to exert anti-inflammatory effects. Acupuncture protects multiple organs from damage during sepsis. Acupuncture can regulate the function of immune cells (such as macrophages and T lymphocytes), inhibit the excessive inflammatory response in sepsis and anti-oxidative stress, protect mitochondrial function, reduce cell apoptosis, and reduce tissue or organ damage. Among these, the Nrf-2/HO-1, PI3K/Akt/Nrf2/HO-1, JAK1/STAT3, SIRT1/NF-κB, HO-1/PINK1, calpain-2/STAT3, and PICK1-TLR4 signalling pathways may be involved in the effects of acupuncture on the inhibition of the inflammatory response, reduction of oxidative stress, and protection of mitochondrial damage in the target organs of sepsis (**Figure 2**).

## 9 Discussion

This article reviews the existing evidence on the use of acupuncture and moxibustion in the prevention and treatment of sepsis. First, mechanistic research has focused on target organs, and the upstream pathway, especially the central integration mechanism, has been less studied. Second, a large number of existing studies on the protective effects of acupuncture on septic organs are related to anti-inflammation, and there are few basic studies on acupuncture and immunosuppression, which cannot clearly explain the improvement and potential mechanism of acupuncture and moxibustion in immunosuppression. Third, clinical sepsis is usually treated after the disease onset. Animal studies have shown that both pre- and post-treatment with acupuncture can treat sepsis, but whether the signal transduction mechanisms involved are similar or different remains unknown. Currently, there are few research reports in this area, and they are fragmented; therefore, the principles cannot be systematically revealed. Fourth, it is difficult to fully replicate the complexity of human sepsis, which is affected and restricted by many factors, in clinical practice. Most patients with clinical sepsis are over 50 years old, whereas the general age of experimental rats is 2-3 months, which is equivalent to 10 years in humans, Turnbull et al. (150) confirmed the correlation between age and mortality in the CLP model. Presently, research mostly uses young and healthy animals without complications for modelling, which is difficult to match with the actual situation in which clinical patients are often old and have multiple complications. In addition, there is a big difference between clinical practice and the fact that no other supportive treatment measures are provided in modelling research. Deitch believed that the selection of models depends on the main objectives of the proposed research and the clinical situation to be modelled (151, 152). Therefore, it is necessary to improve animal models and conduct clinical studies to explore the efficacy of EA in sepsis.

While focusing on the advantages of acupuncture in the prevention and treatment of sepsis, its adverse effects cannot be ignored. The increase in adverse events associated with acupuncture is closely related to the vigorous development of acupuncture and moxibustion. Adverse reactions and accidents associated with acupuncture mainly include dizziness, broken needles, bent needles, stuck needles, infection, and organ injury (35, 44). Adverse reactions to acupuncture are mainly caused by dizziness, unknown allergy history, emotional instability, physical differences, and other reasons. There are also many reasons leading to acupuncture accidents, including a lack of strict disinfection, improper acupuncture operation, improper treatment after acupuncture, bending needles, and folding needles. Differences in the methods of the acupuncturists will cause significant differences in the odds of adverse acupuncture events. For example, some acupuncturists prefer deep stimulation, strong stimulation, or application of needles in dangerous areas to maximise efficacy, which may lead to an increased incidence of adverse acupuncture events. Therefore, acupuncture adverse events can only indicate the safety level of the corresponding acupuncture operator and cannot be used to evaluate the safety of acupuncture therapy as a whole.

A comprehensive understanding of the mechanisms of action of acupuncture and moxibustion in the prevention and treatment of sepsis is important for future animal and clinical studies. Presently, to prevent COVID-19 patients from becoming septic, those who are qualified for asymptomatic infection can undergo EA at Guanyuan, ST36, and other acupoints according to their conditions at home and during rest time to adjust their autoimmunity. The early use of acupuncture and other supplementary anti-inflammatory therapies can effectively prevent the risk of sepsis caused by ‘cytokine storms’ resulting from excessive release of proinflammatory cytokines, and reduce the use of hormones. However, acupuncture has not been widely used for the prevention and treatment of COVID-19, which may be due to a lack of extensive understanding of the anti-inflammatory mechanism of acupuncture. After discharge, some cured cases are accompanied by coughing, poor food intake, fatigue, or abnormal mood, and different degrees of tissue or organ function damage. During this period, the patient’s immunity was low. If they were reinfected with bacteria or viruses, the damage would exacerbate. Therefore, prevention of disease recurrence is necessary. In summary, this article showed that acupuncture can improve the immune function of patients, reduce possible multiple organ dysfunction problems, and further improve the symptoms of discharged patients through targeted acupoint selection. In short, acupuncture may have beneficial effects that can prevent diseases before they occur, worsening of diseases, and their recurrence after rehabilitation.

This review provides strong evidence for the effectiveness of acupuncture and moxibustion in the prevention and treatment of sepsis. Clarification of the existing evidence on acupuncture and moxibustion in the prevention and treatment of sepsis will provide various opportunities for acupuncture, moxibustion, and these two therapies combined with drugs for the prevention and treatment of sepsis. Simultaneously, with interdisciplinary cooperation and the combination of modern science, technology, and equipment, more in-depth and comprehensive research on the roles of acupuncture and moxibustion in preventing sepsis and protecting organs will be promoted. In the future, acupuncture and moxibustion could accurately drive different neural pathways to treat specific diseases. Therefore, it is extremely important to continue research on this subject.

## Author contributions

LY and XL: concept design and manuscript writing. JC, FS and JZ: data collection and analysis. ZC, SZ, and BC made language modifications and reviewed the manuscript. DZ and YG: Concept design and manuscript review. All the authors contributed to the manuscript and approved the submitted version.

## Glossary

**Table:**

MODS	multiple organ dysfunction syndrome
PAMP	pathogen-associated molecular pattern
DAMP	damage-associated molecular pattern
PRR	pattern recognition receptor
TGF-beta	transforming growth factor beta
TNF-α	tumor necrosis factor-α
IL-1beta	interleukin-1beta
Th2	type 2 helper T
HLA-DR	human leukocyte antigen-DR
MDSCs	myeloid-derived suppressor cells
NK	natural killer
LPS	lipopolysaccharide
NOS	nitric oxide synthase
NADPH	nicotinamide adenine dinucleotide phosphate
NOX4	NADPH oxidase 4
PC	protein C
DIC	disseminated intravascular coagulation
TF	tissue factor
TFPI	tissue factor pathway inhibitor
t-PA	tissue-type plasminogenactivator
PAI-1	plasminogen activator inhibitor-1
NETs	neutrophil extracellular traps
EA	electroacupuncture
ST36	Zusanli
ST37	Shangjuxu
PCT	procalcitonin
IFABP	intestinal fatty acid binding protein
TEAS	transcutaneous electrical acupoint stimulation
L: M	lactulose to mannitol ratio
CRP	C-reactive protein
NSE	neuron-specific enolase
SAE	sepsis-associated encephalopathy
APACHE	acute physiologic and chronic health evaluation
CV4	Guanyuan
CLP	cecal ligation and puncture
CASP	colon ascendens stent petitonitis
BL13	Feishu
GV20/DU20	Baihui
ST25	Tianshu
LI11	Quchi
PC6	Neiguan
NPY	neuropeptide Y
MA	manual acupuncture
HSN	heat sensitive neurons
CB2R	cannabinoid CB2 receptor
CAP	Cholinergic anti-inflammatory pathway
α7nAChR	α7 nicotinic acetylcholine receptors
Ach	acetylcholine
CK-MB	creatine kinase MB
NO	nitric oxide
MPO	myeloperoxidase
BL32	Ciliao
LI4	Hegu
DVC	the dorsal vagal complex
AP	acute pancreatitis
NEI	the nerve endocrine immune system
ALI	acute lung injury
MDA	malondialdehyde
SOD	superoxide dismutase
HO-1	heme oxygenase-1
W/D	wet/dry
iNOS	nitric oxide synthase
Nrf2	nuclear factor erythroid-2 related factor-2
ARE	antioxidant response element
ERK1/2	extracellular signal regulated kinases1/2
SIRT1	sirtuin 1
ACE2	angiotensin-converting enzyme 2
RAS	the renin-angiotensin system
AT II	alveolar type II cell
SAE	sepsis-associated encephalopathy
ChAT	choline acetyltransferase
AChE	acetylcholinesterase
eNOS	endothelial nitric oxide
Aβ	β–peptide
H2O2	hydrogen peroxide
CAT	catalase
GSH	glutathione
TJ	tight junction
sIgA	secretory immunoglobulin A
SAKI	sepsis-associated acute kidney injury
SDH	succinate dehydrogenase
ALP	alkaline phosphatase
DU26	Renzhong
PI3K	phosphoinositide 3-kinase
BUN	blood urea nitrogen
Cr	creatinine
NAG	N-acetyl-glucosaminidase
ALT	alanine aminotransferase
AST	aspartate aminotransferase
LDH	lactate dehydrogenase
G-6-Pase	Glucose-6-phosphatase
5’ –Nase	5’- Nucleotidase
XOD	xanthine oxidase
